# Sleep and Cognitive Performance: Exploring Benzodiazepine Receptor Agonist Use, Insomnia Symptoms, and Obstructive Sleep Apnea

**DOI:** 10.1007/s12126-026-09684-6

**Published:** 2026-10-29

**Authors:** Lydia Ann, Tue Te, Michael Mitchell, Cathy Alessi, Jennifer Martin, Joseph M. Dzierzewski, Karen Josephson, Saadia Naeem, Erin Der-Mcleod, Sara Ghadimi, Michelle Zeidler, M. Safwan Badr, Alison Moore, Constance H. Fung

**Affiliations:** 1 University of California Irvine Psychiatry and Human Behavior, Orange, United States; 2 VA Greater Los Angeles Healthcare System, North Hills, United States; 3 VA Greater Los Angeles Healthcare System, North Hills, United States; 4 University of California, Los Angeles, Los Angeles, United States; 5 National Sleep Foundation, Washington, United States; 6 Wayne State University, Detroit, United States; 7 John D. Dingell VA Medical Center, Detroit, United States; 8 University of California, San Diego, San Diego, United States

**Keywords:** Cognition, Sleep, Benzodiazepine, Sleep apnea, Insomnia

## Abstract

**Objectives:**

We hypothesized that benzodiazepine/z-drug (BZRA) dose, insomnia symptoms, and obstructive sleep apnea (OSA) risk are associated with cognitive performance above and beyond other modifiable dementia risk factors.

**Methods:**

In this cross-sectional study, we used baseline data from recruited patients without dementia from one VA site and one academic site to analyze regression models based on previously published risk factors for cognitive impairment, adapted to include sleep and benzodiazepine usage interactions. Changes in R^2^ and model fit (F-tests) were examined for four nested regression models predicting Mini-Mental State Examination (MMSE), Trail Making Test (TMT)-A, TMT-B, and Digit Symbol Substitution Test (DSST). Base Models included common dementia risk factors. Model-2 = Base + Insomnia Severity Index; Model-3 = Model-2 + dose; Model-4 = Model-3 + OSA risk; and Model-5 = Model-4 + OSA-BZRA.

**Results:**

R^2^ for the base model predicting MMSE was 0.139 (n = 313), 0.241 for TMT-A (n = 298), 0.229 for TMT-B (n = 287), and 0.189 for DSST (n = 285). Model-5 R^2^ was 0.142 for MMSE, 0.246 for TMT-A, 0.238 for TMT-B, and 0.190 for DSST, with all F-test p-values >.145.

**Conclusions:**

Sleep disorder variables did not significantly explain additional variation in cognition performance. Studies with a wider range of cognitive performance are needed.

**Supplementary Information:**

The online version contains supplementary material available at https://doi.org/10.1007/s12126-026-09684-6.

## Introduction

The number of people living with cognitive impairment or dementia is expected to rise from 55 million in 2019 to 139 million in 2050 (International AD, 2023). Thus, there has been an increasing focus on addressing risk reduction for dementia by specifically targeting modifiable risk factors for cognitive impairment (less education, hearing loss, untreated vision loss, high LDL cholesterol, smoking, obesity, depression, physical inactivity, diabetes, excessive alcohol consumption, traumatic brain injury, air pollution, and social isolation) described in the Lancet Commission 2024 update (Livingston et al., 2024). Sleep disorders are also recognized to have a bidirectional relationship with cognitive decline.

As a whole, sleep disturbance has been linked to cognitive decline and increased risk of Alzheimer’s disease dementia through pathways such as increasing beta-amyloid burden and amyloid-driven tau phosphorylation, potentially by processes such as increasing systemic inflammation and oxidative stress and reducing glymphatic functioning (Bubu et al., 2020; Irwin & Vitiello, 2019). A common causes of sleep disturbance is obstructive sleep apnea (OSA), which impairs attention, verbal memory, executive function, and learning (Davies & Harrington, 2016). Insomnia is associated with impairment in working memory, episodic memory, and some aspects of executive functioning in some but not all studies (Dzierzewski et al., 2018). Pharmacological treatment for insomnia (i.e. hypnotics such as benzodiazepines, non-benzodiazepine receptor agonists) has been associated with cognitive impairment, described as deficits in memory, learning, attention, and visuospatial ability, as well as major cognitive decline and dementia (Atkin et al., 2018; Markota et al., 2016; Reimers et al., 2025). There is also evidence that mild memory impairment experienced by individuals with short term, low dose benzodiazepine use is dose-dependent and reversible with benzodiazepine discontinuation (Salzman, 2020). These associations are clinically relevant because in clinical settings, they are targeted as potential reversible risk factors for cognitive impairment.

Though the effects of various sleep conditions and their treatments on cognition have been examined in dyads (e.g., hypnotic-cognition, OSA-cognition), patients often have multiple coexisting sleep-related pathologies (e.g., OSA and insomnia) that can impair cognition. One study found that patients with comorbid OSA and insomnia are more likely to self-report cognitive decline compared to patients with just one of the conditions (i.e., OSA only, insomnia only) (Huang et al., 2025). Another study found that individuals with both OSA and insomnia have worse verbal memory performance than individuals with OSA only (Holloway et al., 2025). The combination of insomnia and increased risk of OSA based on a STOP-Bang questionnaire is associated with mild cognitive impairment (Mayer et al., 2023). These studies examined multiple sleep-related risk factors simultaneously as co-occurring risk factors for cognitive impairment rather than each condition’s contribution to cognitive impairment. The latter approach may inform clinical management, particularly regarding whether to prioritize diagnostic sleep evaluation and treatment over non-sleep disorder dementia risk factor management. Further complicating guidance are results from a recent study that found that cognitive performance worsened following behavioral sleep treatment in patients with co-existing insomnia and OSA (Turner et al., 2023).

In this study, we sought to examine the associations of hypnotic use, insomnia symptoms, and OSA risk on cognitive performance in the context of other risk factors for dementia. Using baseline assessment data from a hypnotic deprescribing clinical trial that enrolled adults aged 55 years and older, we hypothesized that higher hypnotic dosing (benzodiazepine-equivalent milligrams) would be associated with lower cognition as measured by Mini-Mental State Examination (MMSE) and Trails A and B performance, above and beyond other contributors to dementia (e.g., vascular risk factors) (Hypothesis A). Our secondary hypothesis was that higher OSA risk would also be associated with lower MMSE and Trails A and B performance, above and beyond other risk factors including hypnotic dose (Hypothesis B). Lastly, we hypothesized that higher hypnotic dose in individuals with OSA would be associated with lower cognition as measure by MMSE and Trials A and B performance than for individuals without OSA (Hypothesis C).

## Methods

### Conceptual Model

We identified dementia risk factors from the Lancet Commission’s 2020 report (Livingston et al., 2020) such as less education, traumatic brain injury (TBI), hypertension, alcohol >= 21 units per week, obesity, smoking, depression, social isolation, physical inactivity, air pollution, diabetes. We then adapted this model to investigate insomnia symptoms, benzodiazepine usage, and OSA risk as additional risk factors to cognitive impairment (Bubu et al., 2017). Our adapted model was also informed by published worked on COMISA (Turner et al., 2023).

### Overview

We used baseline data from a hypnotic deprescribing clinical trial (NCT03687086) (Fung et al., 2024) that recruited patients without dementia from one VA site and one academic site who were taking one of more of the following 5 benzodiazepine or z-drugs (BZRA; alprazolam, clonazepam, lorazepam, temazepam, or zolpidem). Individuals underwent preliminary telephone screening to assess preliminary eligibility. Those eligible underwent informed consent, and two-part baseline face to face assessments were performed in person or via video conferencing with both audio and visual components.

Baseline data gathered during telephone screen and face to face assessments were used in this analysis. These data informed eligibility for randomization for parent trial. Inclusion and exclusion criteria for the hypnotic deprescribing clinical trial are noted in supplemental Table 1. This clinical trial was approved by our local institutional review boards and monitored by a safety officer and a data and safety monitoring board, and there were no ethical concerns.

### Measures

The following independent variables from baseline self-report were used in our base model: age, sex, ethnicity, race, education; vascular risk (> = 1 diagnosis: hypertension, obesity, diabetes, stroke), depression diagnosis, and TBI/head injury; and excessive alcohol use (11 + weekly drinks versus < 11). Sleep variables were Insomnia Severity Index score, BZRA dose (diazepam-equivalent milligrams) based on self-reported 7-day medication log that was informed by concurrent 7-day sleep diary, and OSA risk (3 categories: 1) having a prior diagnosis of OSA and/or using positive airway pressure therapy (PAP), 2) no prior diagnosis of OSA however STOP-BANG score > 4, and 3) no prior diagnosis of OSA and STOP-BANG score <= 4 (Chung et al., 2016). If participants had a prior diagnosis of OSA, their apnea hypopnea index (AHI) from a prior polysomnography was extracted from their medical record. Participants categorized into the first or second category were considered high risk for OSA.

Our dependent variables to evaluate cognitive performance included MMSE (range 0–30) measuring orientation, memory, attention, and language (Folstein et al., 1975), Trail Making Test (TMT) Parts A and B assessing attention, visual search and scanning, sequencing and shifting, psychomotor speed, abstraction, flexibility, executive function, and ability to maintain two trains of thought simultaneously, and Digit Symbol Substitution Test (DSST) reflecting coordination and psychomotor operations. TMT A and B were scored based on time for completion (Corrigan & Hinkeldey, 1987). DSST were scored on the number of correct symbols copied within an allotted time (Wechsler, 1939).

### Statistical Analysis

Descriptive statistics (means and SDs for continuous variables; frequencies and percentages for categorical variables) were computed for selected variables, as shown in Table 1.

**Table 1:** Sample characteristics and demographics (n = 348)

Age (years), Mean ± SD	68.8 ± 8.7
Gender, n (%)	
Male	250 (71.8%)
Female	98 (28.2%)
Ethnicity, n (%)	
Not Hispanic	313 (89.9%)
Hispanic	35 (10.1%)
Raceᵃ	
White	261 (75.0%)
African American	44 (12.6%)
American Indian/Alaska Native	6 (1.7%)
Asian	10 (2.9%)
Hawaiian/Pacific Islander	3 (0.9%)
Missing	24 (6.9%)
Years of Education, Mean ± SD	15.8 ± 2.8 N = 348
Vascular risk, n (%)	
No	114 (32.8%)
Yes	233 (67.0%)
Missing	1 (0.3%)
Depression, n (%)	
No	165 (47.4%)
Yes	182 (52.3%)
Missing	1 (0.3%)
Traumatic brain injury/head injury, n (%)	
No	318 (91.4%)
Yes	28 (8.0%)
Missing	2 (0.6%)
Alcohol Use 11 + weekly drinks, n (%)	
No	279 (80.2%)
Yes	56 (16.1%)
Missing	13 (3.7%)
Insomnia Severity Index, mean total score ± SD	14.2 ± 6.6 N = 348
Diazepam equivalent, 7-day sum, mean ± SD	29.5 ± 31.4 N = 334
OSA High Risk: StopBang >= 5 or AHI >= 5, n (%)	
No	178 (51.1%)
Yes	153 (44.0%)
Missing	17 (4.9%)
MMSE Total score, Mean ± SD	28.2 ± 1.7 N = 346
Trails A time (seconds), Mean ± SD	48.2 ± 25.3 N = 326
Trails B time (seconds), Mean ± SD	130.2 ± 85.2 N = 313
Digit symbol substitution score, Mean ± SD	8.0 ± 2.5 N = 303

MMSE = Mini-Mental State Examination; STOP-BANG = Assessment score for diagnosing Obstructive Sleep Apnea

For each outcome, we fit five regression models which we named Model 1 to Model 5, with Models 3, 4, 5 corresponding to hypotheses A, B, and C, respectively. Model 1 was our base model, including age, sex, ethnicity, race, educational level, presence of a vascular risk factor, depression diagnosis, diagnosis of TBI/head injury, and alcohol use. These variables were selected based on the Lancet commission list of risk factors and the availability of the variables in our data set. Model 2 was our base model + Insomnia Severity Index. We opted to include Insomnia Severity Index as Model 2 rather than as risk factor in Model 1, because of the literature demonstrating inconsistent associations with cognitive impairment. Model 3 was Model 2 + BZRA hypnotic dose, which has been associated with cognitive impairment (a test of hypothesis A). Model 4 was Model 3 + OSA risk, which has also been associated with cognitive impairment (a test of hypothesis B). Model 5 was Model 4 + OSA-BZRA interaction term, to test the combination of OSA risk and BZRA (a test of hypothesis C). We compared Model 2 versus Model 1 using the comparison of nested regression models, yielding the R^2^ for each model, the ∆R^2^ (change in R^2^, Model 2 versus Model 1), the *F*-test of the ∆R^2^ (testing the null hypotheses that ∆R^2^ equals 0), and the corresponding *p* value. The same technique was used for comparing subsequent models (hypothesis A–Model 2 versus 3; hypothesis B–Model 3 versus 4; and hypothesis C–Model 5 versus 4). A priori power calculations showed that using sample size of 200 with alpha = 0.05 yielded 86% power to detect a change in R^2^ of 0.04 using a base value of R^2^ of 0.10 (that is, an increase from 0.10 to 0.14).

All analyses used alpha = 0.05 and were performed using Stata version 18.0.

## Results

Of 348 participants (*m*_age_ 68.8, 28% female, *m*_education_ 15.8), 313 were included for MMSE (*m* = 28.2), 298 for TMT-A (*m* = 48.2 s), 287 for TMT-B (*m* = 130.2 s), and 285 for DSST (*m* = 8.0) analyses. Mean ISI was 14.2 (SD 6.6). Mean 7-day BZRA total dose was 29.5 diazepam equivalents (SD 31.4). 44% were high risk for OSA. Additional sample characteristics are presented in Table 1.

The Model 1 R^2^ was 0.139 for MMSE with n = 313, 0.241 for TMT-A with n = 298, 0.229 for TMT-B with n = 287, and 0.189 for DSST with n = 285. For MMSE, the ∆R^2^ values for ranged from 0.000 to 0.002 (*p*s > 0.387). For TMT-A the ∆R^2^ values ranged from 0.000 to 0.002 (*p*s > 0.357). For TMT-B the ∆R^2^ values ranged from 0.000 to 0.006 (*p*s > 0.145). For DSST, the ∆R^2^ values ranged from 0.000 to 0.001 (ps > 0.67 s). The ∆R^2^ for Models 2 versus 1, 3 versus 2 (hypothesis A), 4 versus 3 (hypothesis B), and 5 versus 4 (hypothesis C) for all cognitive performance metrics were not statistically significant (*p*s > 0.145), which suggest that the addition of BZRA, OSA-risk, and the interaction term for OSA risk-BZRA did not significantly improve model fit beyond Model 1, the model that lacked sleep variables, and thus do not confer additional explanatory value for MMSE, TMT-A, TMT-B, or DSST above and beyond the combination of variables in the base model (age, sex, ethnicity, race, educational level, presence of a vascular risk factor, depression diagnosis, diagnosis of TBI/head injury, and alcohol use).

**Fig. 1:**
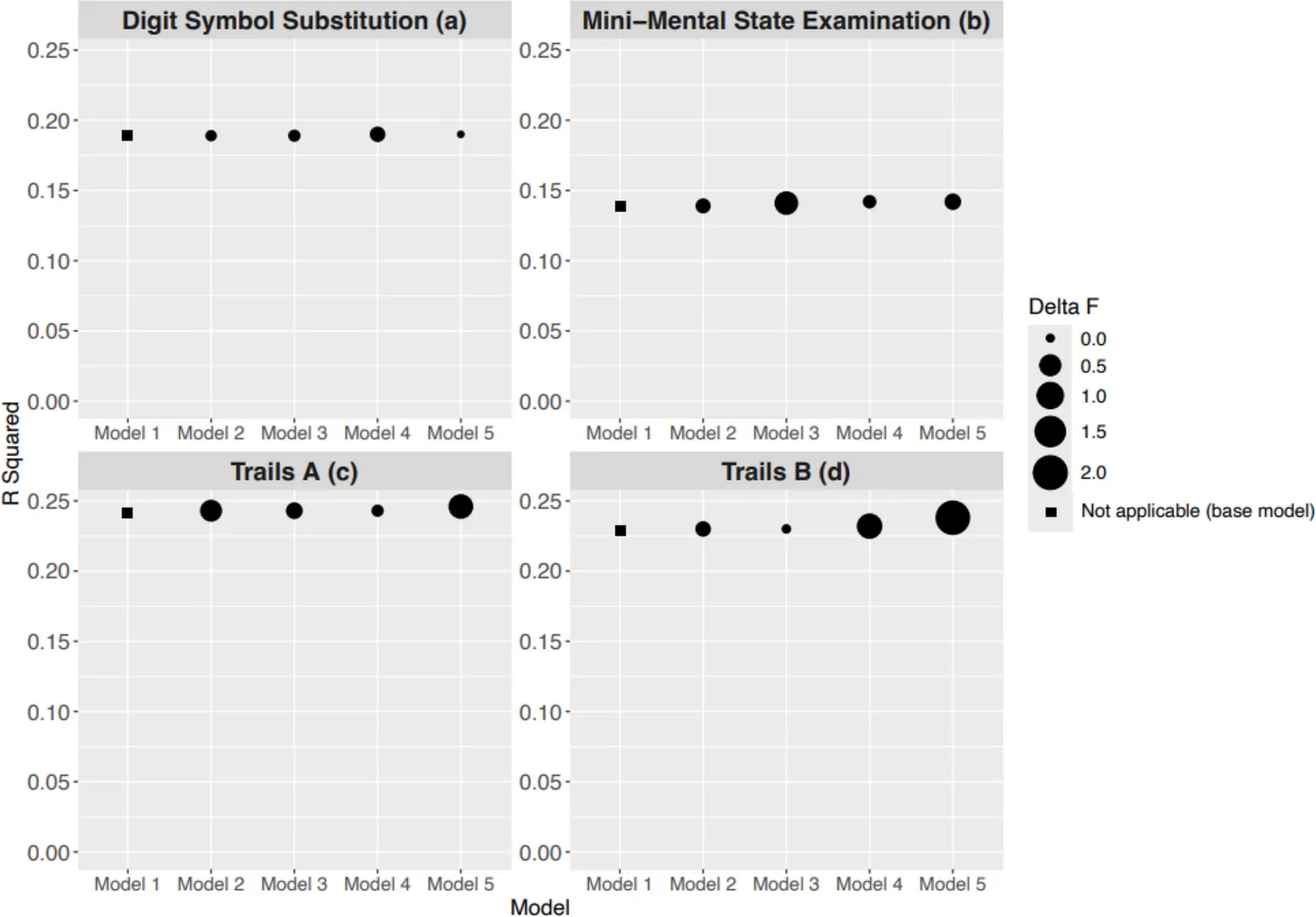
R-plot of cognitive testing across Models 1–5

Figure 1 summarizes the results of the nested regression models.

## Conclusions

We examined associations between insomnia symptom severity, hypnotic dose, OSA risk, and cognitive performance, above and beyond other dementia risk factors including educational level, vascular risk factors, depression, TBI/head injury, and excessive alcohol use. While we found that more than 13% of variation in cognitive performance is explained by a set of nine well-described risk factors (Livingston et al., 2020), model performance did not improve significantly when each sleep-related factor was added. Insomnia symptoms, BZRA dose, OSA risk, and the interaction between BZRA dose and OSA risk did not significantly explain additional variation in cognitive performance above the cumulative variance explained by the set of baseline risk factors.

To our knowledge, no prior studies have examined in this manner the incremental role of sleep-related factors with relationship to other dementia risk factors and with respect to one another (e.g., hypnotic versus OSA risk). By using nested regression, we were able to examine the incremental contribution of sleep-related risk factors rather than the risk associated with simultaneous/co-occurring risk factors. This approach provides an empirical basis for a common scenario–for patients already managing multiple medical conditions in which addressing every risk factor simultaneously may not be feasible or preferred. Our findings seemingly contradict recent research associating sleep disorders with cognitive impairment performance (as described in the Introduction section), but may be more a reflection of the relative roles of overlapping pathways or shared risk factors for cognitive impairment between our base model variables and sleep-related variables (that is, the sleep-related variables may not be exerting independent, additive effects) in a relatively well-educated (average of 15.8 years of education) and cognitively intact (MMSE average 28.2 ± 1.7) population.

One notable limitation of our study is the generalizability of our sample, as our participants were limited to those already prescribed hypnotics and only 28% of our participants were female. We were unable to incorporate PAP adherence information. Lastly, we used the MMSE, which is less sensitive than tests such as the Montreal Cognitive Assessment in detecting early cognitive impairment.

In conclusion, our study examined the contributions of sleep disorder variables above and beyond common dementia risk factors to inform diagnostic and treatment pathways for addressing cognitive functioning in individuals with co-existing sleep conditions. While the common dementia risk factors accounted for a significant degree of variation in cognitive performance, none of our models assessing insomnia, hypnotic use, OSA risk, and the interaction between hypnotic use and OSA risk explained additional variation. Future research could involve comparing cognition in a population taking and not taking medications for insomnia and comparing across a broader population with more varied cognitive status. In particular, assessing these relationships in younger patients (i.e. 45–65 yo) with mild cognitive impairment would be an interesting exploration into age of onset, and impact on cognitive status, and potential reversibility of cognitive changes.

## Supplementary Information

Below is the link to the electronic supplementary material.

Supplementary file1 (DOCX 18 KB)

## Data Availability

The data that support the findings of this study are available from the Veterans Affairs Administration, but restrictions apply to the availability of these data, so are not publicly available. The data are, however, available upon request and with the permission of the Veterans Affairs Administration.
