# Supplementary material for: Sleep and Cognitive Performance: Exploring Benzodiazepine Receptor Agonist Use, Insomnia Symptoms, and Obstructive Sleep Apnea
Source: Ageing Int. 2026 Oct 29;51(4):48. doi: 10.1007/s12126-026-09684-6 (PMC13719709; doi:10.1007/s12126-026-09684-6)
Supplement: Supplementary file 1 [file 12126_2026_9684_MOESM1_ESM.docx]

**Supplemental Table 1: Enrollment Criteria for Parent Trial**

Inclusion Criteria

Age >= 55 years

Use of lorazepam, alprazolam, clonazepam, temazepam, and/or zolpidem^b^ for current or prior insomnia

symptoms 2 or more nights per week for at least 3 months (based on a combination of self-report and

chart review)^c^

Current or prior insomnia symptoms (based on self-report)

Available to attend weekly in-person sessions over 9 weeks.

Exclusion Criteria^a^

*High risk for complications in outpatient hypnotic discontinuation program:*

Seizure disorder (based on self-report and chart review)

Supratherapeutic or high baseline hypnotic dose (> diazepam-equivalent of 8 mg/night based on a combination of medication log and chart review)

High baseline risk of complicated withdrawal; benzodiazepine intoxication or current or past symptoms of complicated benzodiazepine/alcohol withdrawal (e.g., seizure, delirium at baseline (prior to taper))

(based on self-report and chart review)

Polydrug use (e.g., chronic high dose opioids) (based on self-report including the World Health

Organization Alcohol, Smoking, and Substance Involvement Screening Test and chart review)

Unable to keep study medications in secure location (based on self-report and chart review)

Evidence of prescription fraud (e.g., multiple prescriptions for same drug filled at multiple pharmacies during overlapping time periods, diversion) (based on chart review, state drug monitoring program)

*Discontinuation of hypnotic not appropriate:*

Study-targeted hypnotic used to treat another clinical condition (e.g., panic disorder) (based on self-report, chart review, and feedback from prescribing clinician)

*Poor candidate for cognitive behavioral therapy for insomnia:*

Presence of bipolar disorder (based on combination of self-report, chart review, and Mini International Neuropsychiatric Interview for individuals with unclear diagnoses)

Cognitive impairment (e.g., Mini-Mental State Examination < 24)

Sleep/wake difficulty is better explained by another sleep disorder such as restless legs syndrome, narcolepsy, insufficient sleep syndrome, or circadian rhythm sleep-wake disorders (based on screening with Morningness-Eveningness Questionnaire, assessment for restless legs syndrome, chart review).

Untreated sleep-disordered breathing (respiratory event index >= 15 and < 30 plus excessive daytimes sleepiness, or respiratory event index >=30) (based on STOP-BANG plus home sleep apnea test or in-lab polysomnography)

Medically/psychiatrically unstable (e.g., planned major surgery during the study period; psychosis, suicidal, active alcohol/substance abuse based on history and medical records) (based on self-report,

World Health Organization Alcohol, Smoking, and Substance Involvement Screening Test and chart

review)

Unstable housing situation (based on self-report and chart review)

------------------------------------------------------------------------------------------------------------------------------------------

^a^Inclusion and exclusion criteria were reviewed for each prospective participant prior to randomization during a team meeting to determine eligibility.

^b^Zolpidem was the most frequent z-drug prescribed at the VA site due to formulary restrictions. Other z-drugs were not included because the compounding pharmacy that initially agreed to supply the medications indicated that it would only agree to a limited number of drug preparations (ultimately, a different compounding pharmacy was contracted but all of the regulatory submissions had already been approved).

^c^Initially, patients were required to take the target medication(s) more than 3 nights per week, but a change was made to the protocol after recruitment started to include patients taking the medication 2 or more nights per week

^d^8 milligram diazepam-equivalent estimates were based on values provided by clincalc.com (<https://clincalc.com/Benzodiazepine/>) and GPnotebook (Zolpidem to diazepam equivalence in hypnotic withdrawal – GPnotebook). The maximum dose was alprazolam 0.75 mg/night, clonazepam 0.75 mg/night, lorazepam 1.25 mg/night, temazepam 22.5 mg/night, and zolpidem 12.5 mg/night on average (in some cases, participants took higher doses of their medication on some nights and lower doses on other nights)
